# Persistent Organic Pollutants Induced Protein Expression and Immunocrossreactivity by *Stenotrophomonas maltophilia* PM102: A Prospective Bioremediating Candidate

**DOI:** 10.1155/2013/714232

**Published:** 2013-06-26

**Authors:** Piyali Mukherjee, Pranab Roy

**Affiliations:** ^1^Department of Biotechnology, University of Burdwan, Golapbag More, Burdwan, West Bengal 713104, India; ^2^Department of Biotechnology, Haldia Institute of Technology, Haldia, West Bengal 721657, India

## Abstract

A novel bacterium capable of growth on trichloroethylene as the sole carbon source was identified as *Stenotrophomonas maltophilia* PM102 by 16S rDNA sequencing (accession number of NCBI GenBank: JQ797560). In this paper, we report the growth pattern, TCE degradation, and total proteome of this bacterium in presence of various other carbon sources: toluene, phenol, glucose, chloroform, and benzene. TCE degradation was comparatively enhanced in presence of benzene. Densitometric analysis of the intracellular protein profile revealed four proteins of 78.6, 35.14, 26.2, and 20.47 kDa while the extracellular protein profile revealed two distinct bands at 14 kDa and 11 kDa that were induced by TCE, benzene, toluene, and chloroform but absent in the glucose lane. A rabbit was immunised with the total protein extracted from the bacteria grown in 0.2% TCE + 0.2% peptone. Antibody preadsorbed on proteins from peptone grown PM102 cells reacted with a single protein of 35.14 kDa (analysed by MALDI-TOF-mass-spectrometry) from TCE, benzene, toluene, or chloroform grown cells. No reaction was seen for proteins of PM102 grown with glucose. The PM102 strain was immobilised in calcium alginate beads, and TCE degradation by immobilised cells was almost double of that by free cells. The beads could be reused 8 times.

## 1. Introduction

Many substances with toxic properties have been brought into the environment through human activity. These substances vary in level of toxicity and danger to human health [1]. Trichloroethylene, mainly used as a metal degreasing solvent, a suspected carcinogen [2], and a USEPA priority pollutant, is the most commonly reported contaminant of groundwater at hazardous waste sites [3]. Toluene is a common solvent, able to dissolve paints, paint thinners, and silicone sealants [4]. Toluene is, however, much less toxic than benzene and has, as a consequence, largely replaced it as an aromatic solvent in chemical preparation. Benzene is a known carcinogen, whereas toluene has very little carcinogenic potential [5]. The major use of phenol involves its conversion to plastics or related materials. Phenol, is a strong neurotoxin and if injected into the blood stream it can lead to instant death [6]. The major use of chloroform today is in the production of the chlorodifluoromethane, a major precursor to tetrafluoroethylene, that is used in the production of teflon. The US National Toxicology Program's twelfth report on carcinogen implicates it as reasonably anticipated to be a human carcinogen [7]. 

Conventional methods to remove or reduce toxic substances introduced into soil or ground water via human activities include pump and treat systems, soil vapour extraction, incineration, and containment, which suffer from recognizable drawbacks and may involve some level of risk. Bioremediation is an option that offers the possibility to destroy or render harmless various contaminants using natural biological activity. The cometabolic degradation of TCE and other chlorinated and aromatic solvents such as benzene, toluene, phenol, and chloroform by different bacteria has been extensively studied [8–11]. This research paper highlights how the soil bacterium: *Stenotrophomonas maltophilia* PM102 expresses a single 35.14 kDa protein induced in the presence of not only trichloroethylene but also toluene, benzene, and chloroform. This may be a major defence protein employed by the bacterium to degrade such toxic contaminants. 

Immobilised bacterial cells and enzymes have been used in a variety of scientific, environmental, and industrial applications. Encapsulated cells have numerous advantages over free cells as seen for increased metabolic activity [12], protection from toxic substances [13], and increased plasmid stability [14]. In the present work, the study of the encapsulation of *S. maltophilia* PM102 in calcium alginate beads was implemented. 

## 2. Materials and Methods

### 2.1. Bacterial Isolate

**Figure 1:**
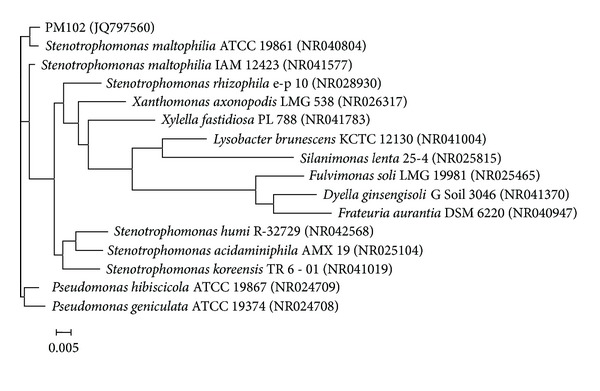
Neighbour joining tree based on PM102 16S rRNA gene sequence comparison with 16S rRNA gene sequences retrieved from the NCBI GenBank, constructed using MEGA 5 software. The numbers in parentheses are GenBank accession numbers (see [15]).

The bacterium *Stenotrophomonas maltophilia* PM102, capable of growing on TCE as the sole carbon source, was isolated from the waste disposal site of the industrial belt lining Asansol and Dhanbad, India, by serial dilution and plating technique. 10 g soil sample was suspended in 100 mL sterile distilled water. The soil particles were allowed to settle down, and 1 mL of the supernatant was transferred to 9 mL sterile distilled water containing 0.9% NaCl. Serial dilutions up to 10^−6^ were performed. 0.1 mL from all tubes was spread on plates containing King's B (KB) medium with TCE (2 *μ*L/mL). The molecular identification of the isolated bacterium was done by 16S rDNA sequencing (universal primers 27F: 5′ AGA GTT TGA TCC TGG CTC AG 3′ and 1492R: 5′ TAC GGY TAC CTT GTT ACG ACT T 3′ were used) that was compared with reported sequences in the Genbank database. Phylogenetic tree was constructed by aligning PM102 sequence with reference sequences obtained from NCBI GenBank (Figure 1).

TCE degradation by the isolate PM102 was studied using Fujiwara test and the release of chloride ions by mineralization [15].

### 2.2. Growth Conditions

 The PM102 strain was grown in 100 mL minimal King's B medium (Na_2_HPO_4_—1 g/L, K_2_HPO_4_—3 g/L, NH_4_Cl—1 g/L, and MgSO_4_·7H_2_O—0.4 g/L), pH 7 with 0.2% of different carbon sources, that is, toluene, phenol, glucose, chloroform, and benzene, each with 0.1% peptone by shaking at 90 rpm at 34°C for 48 hours. Growth of the bacteria was determined by measuring the absorbance at 620 nm.

### 2.3. Fujiwara Test to Determine TCE Degradation in Presence of Other Organic Pollutants

In the Fujiwara test, polychlorinated hydrocarbons in presence of pyridine and alkali give a red colour [16]. PM102 cells harvested from 100 mL culture in minimal medium with 0.2% TCE were suspended in 20 mL phosphate buffer with 0.3% TCE in acetone and 0.2% other carbon sources (toluene, benzene, chloroform, and phenol resp.) and incubated at 34°C at rpm 100. After every 60- minute interval, 2 mL of the cell suspension was taken and treated with 2 mL pyridine and 2 mL 5 N NaOH followed by heating at 80°C for 2 minutes. The experiment was carried out from 0 hours (just after inoculation) to 3 hours. Absorbance of the red aqueous phase was noted at 470 nm.

### 2.4. Protein Extraction

#### 2.4.1. Intracellular Proteins

The cells from 100 mL culture was harvested by centrifugation at 4°C, rpm 10,000 for 10 minutes. Cell pellet thus obtained was suspended in 1 mL solution 1 (10 mM EDTA pH 8, 50 mM glucose, and 25 mM Tris-HCl pH 8) with 200 *μ*L of 10 mg/mL lysozyme, vortexed, and incubated at 37°C for 1 hr followed by 30 minutes incubation at 4°C. Lysozyme extraction gave better cell lysis when followed by temperature shock. The suspension was centrifuged at 10,000 rpm for 10 minutes at 4°C, and the pellet containing cell debris was discarded. Supernatant thus obtained containing the intracellular proteins was stored at −20°C. Concentration of proteins was determined by Bradford's method [17].

#### 2.4.2. Extracellular Proteins

The supernatant obtained from 100 mL culture of PM102 cells in KB medium with 0.2% carbon sources plus 0.1% peptone was treated with 30% TCA and kept at 4°C overnight. The suspension was centrifuged at 12,000 rpm for 10 minutes. The protein precipitate thus obtained was washed thrice with chilled acetone and finally dissolved in 100 *μ*L stacking gel buffer (0.5 M Tris-HCl). Protein concentration was determined by Bradford's method.

### 2.5. Total Protein Profile of PM102

The extracted protein samples were boiled with Laemmli buffer (1% SDS, 5% Mercaptoethanol, 0.05% Bromophenol Blue in 25 mM Tris-HCl, and 10% glycerol, pH 6.8) for 10 minutes and electrophoresed on a 12% gel at 200 volts. Equal amount of protein was loaded in each well, that is, 40 *μ*g intracellular proteins and 30 *μ*g extracellular proteins, in two separate gels. The gel was stained with Coomassie Blue R250 (0.2%) and destained with 30% destainer (30 mL methanol, 7 mL acetic acid, and the rest distilled water to make a total volume of 100 mL). The gel was analysed by image analysis tool of Quantum Capt software in the gel documentation system (Vilber Lourmat, France). 

### 2.6. Immunisation of Rabbit

A rabbit was immunised with the total protein of the bacteria grown in 0.2% TCE + 0.2% peptone. The rabbit was injected subcutaneously at 4 to 5 different sites with this protein homogenate mixed 1 : 1 with Freund's complete adjuvant twice (once per month) followed by two more injections of the same protein homogenate mixed 1 : 1 with Freund's incomplete adjuvant. 

### 2.7. Western Blot

The SDS PAGE gel of the intracellular proteins obtained from cells grown in TCE, toluene, benzene, chloroform, glucose, and peptone, after electrophoresis (unstained), was electroblotted onto nitrocellulose membrane BA85 (Sigma Aldrich, USA) at 45 volts for 3 hours. The nitrocellulose membrane was blocked with 3% milk powder for 1 hour at room temperature and washed thrice with buffer A (10 mM tris HCl pH 8, 1 mM EDTA pH 8, 0.05% tween 20 and 0.9% NaCl), followed by incubation in 1 : 100 dilution of the rabbit antiserum in the same buffer, at 4°C overnight. Then the membrane was washed with buffer A thrice, five minutes each, and incubated in 1 : 15,000 dilution of goat anti-rabbit IgG coupled to alkaline phosphatase (Sigma Aldrich, USA) in buffer A for 2 hours at room temperature. The membrane was again washed in buffer A, 5 minutes each, for three times and equilibrated for 20 minutes in alkaline phosphatase buffer (100 mM tris HCl pH 9.5, 100 mM NaCl, and 5 mM MgCl_2_). The membrane was stained with BCIP/NBT (5-bromo,4-chloro,3-indolyl phosphate/nitroblue tetrazolium) in alkaline phosphatase buffer for colour development within 15 minutes.

### 2.8. Preadsorption of the Serum Antibody

Small strips of nitrocellulose membrane were soaked in peptone grown cellular proteins and air dried. These strips were immersed in 1 : 100 dilution of the antiserum in buffer A and incubated by gentle shaking for 1 hour. This process was repeated until all the antibodies against the common peptone antigens were removed.

### 2.9. MALDI-TOF/MS Analysis

The 35.14 kDa band was excised from the Coomassie blue stained gel, destained with 250 *μ*L 100 mM ammonium bicarbonate/acetonitrile (1 : 1 v/v) for 30 minutes with occasional vortexing. This step was repeated till all of the blue stain got removed. 500 *μ*L 100% acetonitrile was added for 15 minutes till the gel pieces shrunk and became white in colour. Acetonitrile was discarded, and gel pieces were air dried for 10 minutes in room temperature and reswollen in 25 ng/*μ*L Trypsin (Sigma Aldrich, USA). Trypsin digestion was performed in ice for 1 hour followed by overnight incubation at 37°C with mild shaking. The trypsinised peptide solution was taken in a fresh Eppendorf tube and lyophilised for 1 hour. The lyophilised product was dissolved in 5 *μ*L 30% acetonitrile with 0.1% TFA solution. 4.5 *μ*L of the peptide solution was spotted on target plate along with 4.5 *μ*L of CHCA matrix solution and analysed with MALDI TOF Proteomics Analyzer (Applied Biosystems, Darmstadt, Germany). This was carried out at the Indian Institute of Chemical Biology, Kolkata.

### 2.10. Cell Immobilisation

PM102 cells were grown in 200 mL minimal KB medium with 0.2% TCE, for 48 hrs. Cells were harvested by centrifugation at 10,000 rpm, 4°C for 10 minutes. The cells thus obtained were suspended in 3.5 mL 0.05 M Tris-HCl pH 8.5 and mixed with 3.5 mL of 4% sodium alginate solution. This suspension was added dropwise to a magnetically stirred cold solution of 100 mM calcium chloride with a micropipette. All solutions were autoclaved and the entire work was performed under the laminar airflow hood. Cells immobilised in beads of calcium alginate were stirred in the calcium chloride solution for another hour at 20°C. Then the beads were washed thrice in sterile water to remove any traces of chloride and finally suspended in 50 mL solution of 0.05 mM Tris and stored at 4°C. Alginate beads without cells were used as control.

### 2.11. Cell Viability Test

One bead was suspended in 1 mL sterile water in an Eppendorf tube and crushed with a sterile glass rod. This 1 mL suspension was added to 9 mL sterile distilled water and serially diluted to 10^−3^ dilutions. 0.1 mL from each of the dilution tubes was spread on plates containing minimal KB medium with 0.2% TCE and incubated for 72 hrs. The number of colonies formed was counted and total number of PM102 cells present in each bead was calculated by the formula: (The number of colonies in plate × amount of inoculum added)/stage of serial dilution. Cell viability in the immobilised beads was checked after each treatment cycle. 

### 2.12. Monitoring TCE Degradation by Free and Immobilised Cells

 PM102 cells, grown in 200 mL minimal KB medium with 0.2% TCE, were immobilised in calcium alginate. PM102 cells were grown in 200 mL of the same medium and harvested by centrifugation. Free cells thus obtained were used as control in the TCE degradation experiment to determine if cell immobilisation affected the rate of TCE degradation. Mineralisation of TCE to release free chloride by PM102 cells was tested by titration with silver nitrate in the presence of K_2_CrO_4_ as indicator. Initially, a white precipitate of AgCl, is formed when free chloride is present in the suspension but when free chloride is no longer left in the medium, the solution turns reddish brown due to the formation of Ag_2_CrO_4_. 

The free and immobilised PM102 cells were suspended separately in 50 mL 0.05 mM Tris with 0.2% TCE and incubated at 37°C by gentle shaking for 48 hrs. 10 mL of this suspension was centrifuged and the cell pellet was discarded. The supernatant was taken in a flask, and 10 drops of 0.3 M K_2_CrO_4_ was added and titrated against 10 mM AgNO_3_ at 0 hrs (just after suspension) and after 48 hrs. A standard curve was plotted by varying the concentration of NaCl from 3 mM to 15 mM, (Figure 10(a)), from which the concentration of free chloride released after the respective time intervals was calculated. The experiment was done in triplicate and mean values of the readings are given.

## 3. Results and Discussion

### 3.1. Growth in Presence of Different Carbon Sources

**Figure 2:**
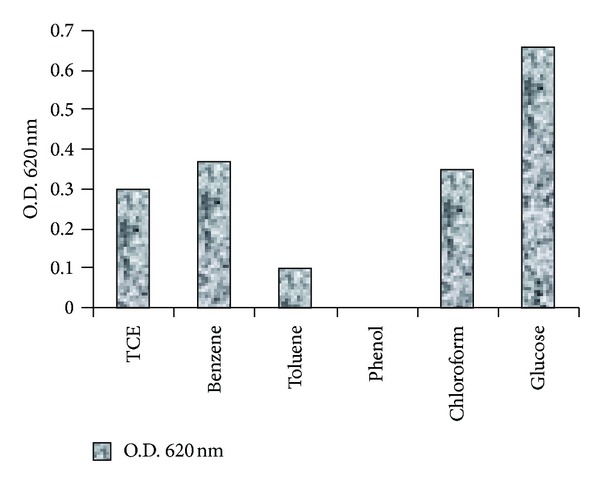
Growth of *S. maltophilia* PM102 in presence of different carbon sources after 48 hrs.

When the PM102 strain was grown in 0.2% of the various carbon sources, TCE, toluene, phenol, glucose, chloroform, and benzene with 0.1% peptone, an interesting growth pattern was observed (Figure 2). No growth was detected in presence of phenol even when 0.1% peptone was present in the medium, indicating that phenol has an inhibitory effect on the bacterium. Growth in presence of TCE, benzene, and chloroform was almost the same, whereas comparatively less growth was seen in presence of toluene. Glucose gave maximum growth and was used as control for the protein profile studies. 

### 3.2. TCE Degradation in Presence of Other Organic Pollutants

**Figure 3:**
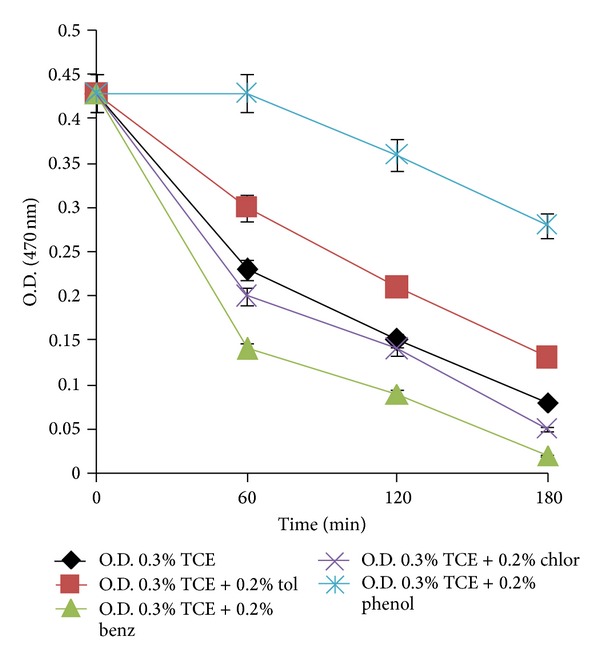
Degradation of 0.3% TCE by PM102 in presence of 0.2% of different carbon sources (toluene, benzene, chloroform, and phenol) as observed by Fujiwara test. Error bars with 5% SEM are displayed.

In Fujiwara test, the intensity of red colour (absorbance at 470 nm) indicates the amount of polychlorinated compound still present in the medium. In the Fujiwara test for TCE degradation by PM102, a decrease in absorbance corresponding to decrease in colour intensity of the aqueous phase with time was noted, thus confirming TCE degradation. This TCE disappearance was more rapid in presence of 0.2% benzene and 0.2% chloroform as compared to that of 0.2% toluene. A marked decrease in TCE degradation rate was noted in presence of 0.2% phenol (Figure 3).

### 3.3. Intracellular Protein Profile of PM102 Grown in Different Carbon Sources

**Figure 4:**
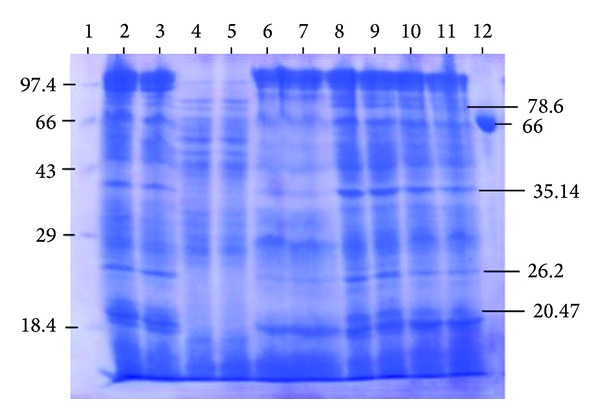
12% SDS PAGE of intracellular proteins of *S. maltophilia *PM102 with different carbon sources. Lane 1 has medium molecular weight marker (kDa). Lanes 2, 3—0.2% TCE + 0.1% pep; lanes 4, 5—0.2% glucose + 0.1% pep; lanes 6, 7—0.2% toluene + 0.1% pep; lanes 8, 9—0.2% chloroform + 0.1% pep; lanes 10, 11—0.2% benzene + 0.1% pep; lane 12—BSA.

**Figure 5:**
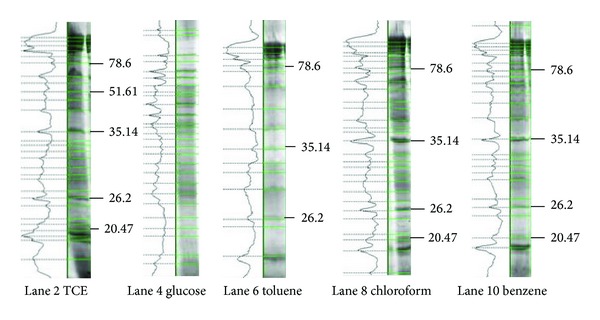
Densitometric analysis of the 12% gel by Quantum Capt software showing the differential expression of proteins under varying culture conditions—0.1% peptone and 0.2% of TCE, glucose, toluene, chloroform, and benzene, respectively.

Figure 4 shows the 12% SDS PAGE of lysozyme extracted intracellular proteins. Densitogram analysis of the above gel (Figure 5) clearly revealed 3 major bands of molecular weights 78.6, 35.14, and 26.2 kDa induced in the presence of TCE, toluene, chloroform, and benzene, separately. These proteins were not present when the PM102 strain was grown in the same medium with glucose as the carbon source. These carbon sources were not given in combination. PM102 cells were grown in KB medium with these carbon sources separately and intracellular proteins were extracted. A 20.47 kDa band was seen for TCE, benzene, and chloroform that was absent in toluene and glucose lanes. Thus, these induced proteins might have a possible role in the degradation of toxic pollutants like TCE, toluene, benzene, and chloroform, although further experiments are required to document the exact mechanism of degradation.

### 3.4. Extracellular Protein Profile of PM102 Grown in Different Carbon Sources

**Figure 6:**
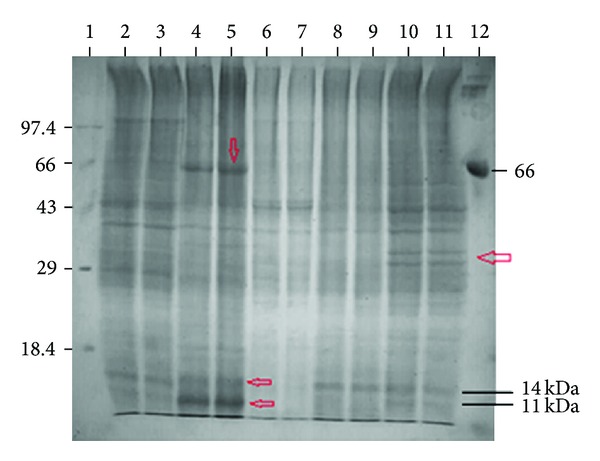
12% SDS PAGE of extracellular proteins of *S. maltophilia *PM102 with different carbon sources. Lane 1 has medium molecular weight marker (kDa). Lanes 2, 3—0.2% TCE + 0.1% pep; lanes 4, 5—0.2% toluene + 0.1% pep; lanes 6, 7—0.2% glucose + 0.1% pep; lanes 8, 9—0.2% chloroform + 0.1% pep; lanes 10, 11—0.2% benzene + 0.1% pep; lane 12—BSA.

From the extracellular protein profile of the bacteria grown with different carbon sources, a major expressed band of 67 kDa and two prominent low molecular weight bands of 14 kDa and 11 kDa were seen in presence of toluene, which were not there in presence of glucose. These bands could be induced in presence of toluene. The low molecular weight bands were also seen in presence of the other carbon sources but were absent in the glucose lane (Figure 6).

### 3.5. Western Blot

**Figure 7:**
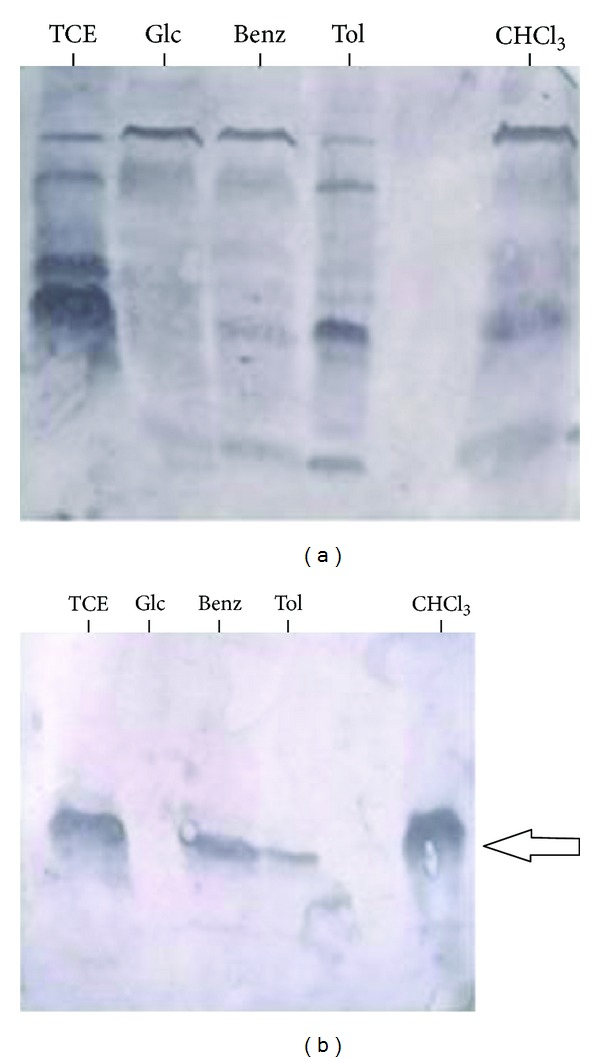
(a) Western blot with total antibody against intracellular proteins of PM102. First lane shows reaction against proteins from PM102 grown in 0.2% TCE + 0.1% peptone; second lane shows total antibody response against proteins extracted from PM102 isolate grown in 0.2% glucose with 0.1% peptone; third lane shows reaction against proteins from PM102 grown in 0.2% benzene and 0.1% peptone; fourth lane shows reaction of total antibody against proteins from PM102 grown with 0.2% toluene + 0.1% peptone; the last lane shows antibody response against proteins of PM102 grown in 0.2% chloroform with 0.1% peptone. (b) Western blot with antibody preadsorbed in proteins from PM102 grown in peptone. The preadsorbed antibody reacted against a single 35.14 kDa protein from PM102 grown with TCE, benzene, toluene, or chloroform. No reaction was seen against proteins from PM102 grown in glucose.

Western blot analysis was carried out with intracellular proteins obtained by lysozyme extraction from PM102 strain grown with different carbon sources. In the western blot analysis with the total antiserum, bands were observed in response to TCE, toluene, benzene, and chloroform grown proteins as well as glucose grown proteins of PM102 (Figure 7(a)). Although several proteins of molecular weights 78.6, 26.2, and 20.47 kDa were induced in presence of the different carbon sources, the 35.14 kDa band was of interest because after the antiserum was preadsorbed on proteins of the PM102 isolate grown in peptone, strong response was obtained only against this single band of 35.14 kDa for TCE, benzene, toluene, or chloroform grown proteins of PM102 while no response for glucose grown proteins from PM102 was seen (Figure 7(b)). 

### 3.6. MALDI-TOF/MS Analysis

**Figure 8:**
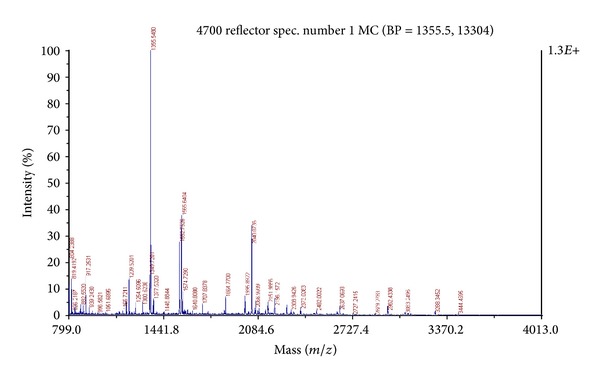
MS/MS spectrum of the 35.14 kDa protein following in gel digestion with 25 ng/*μ*L trypsin.

Unless the genome of the species of interest is completely sequenced, the possible way of identifying a protein is through peptide mass fingerprinting (PMF) [18]. There is no guarantee that the true sequence of the analyte protein is actually present in the database. If it is missing, then high scoring matches from other species are of interest because they are likely to be homologous to the unknown as seen with our protein of interest. Mass spectra of the 35.14 kDa TCE inducible protein are shown in Figure 8. This data was analysed with peptide mass fingerprinting (PMF) platform: The Global Proteome Machine (Global Proteome Machine Organization) and Protein Pilot (Applied Biosystems, Foster City, CA). The PMF data was aligned to 15 proteins with existing PMF data of the group “other proteobacteria” as the PM102 isolate belonged to the class of **γ**proteobacteria. The highest score (60) obtained was with a hypothetical uncharacterised protein of unknown function from *Bacteroides thetaiotaomicron* (UniProtKB/TrEMBL Acc no: Q8A696) (Table 1). 

The proteome of* Bacteroides thetaiotaomicron* includes an elaborate apparatus for acquiring and hydrolyzing otherwise indigestible dietary polysaccharides and an associated environment-sensing system consisting of a large repertoire of extracytoplasmic function, sigma factors, and one- and two-component signal transduction systems [19]. The PM102 strain also has extreme metabolic capabilities as it can grow on trichloroethylene as the sole carbon source and on other persistent organic pollutants in presence of peptone. The complete genome of *Stenotrophomonas maltophilia *K279a has been sequenced and found to contain organic solvent resistant gene (ostA) that encodes for organic solvent tolerance protein besides heavy metal resistance genes [20]. It is a well-known fact that trichloroethylene is used as an organic solvent for oils and resins and metal degreasing purpose [21]. As the PM102 isolate is different from the K279a strain, it may not possess the same protein (PMF data of the 35.14 kDa protein from the PM102 isolate did not match to any of the proteins from K279a in the database records as seen by MASCOT search). Thus, the 35.14 kDa protein seems to be novel without prior record in the database.

### 3.7. Monitoring TCE Degradation by Free versus Immobilised Cells

**Figure 9:**
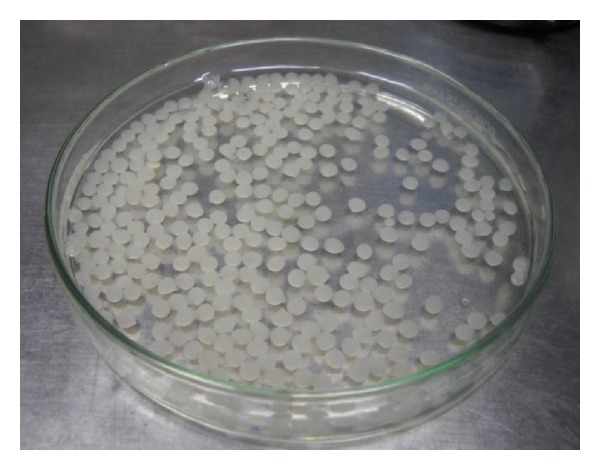
Calcium alginate beads containing immobilised cells of *S. maltophilia* PM102.

The encapsulation of the bacterial cell offers space for the cell growth and good diffusion properties and reusability which provide an extra edge to the encapsulated cells as far as bioremediation is concerned. Initial attempts of cell immobilisation failed causing disruption of the beads within 24 hrs. Initially, PM102 cells were suspended in chloride-free minimal medium: K_2_HPO_4_—3 g/L, Na_2_HPO_4_—1 g/L, (NH_4_)_2_SO_4_—1 g/L, and MgSO_4_·7H_2_O—0.4 g/L. It was assumed that phosphates present in the minimal medium weakened the cross links in the calcium alginate beads, causing bead disruption. Then, immobilisation experiment was entirely carried out by suspending the cells and beads in 0.05 mM Tris, and stability and integrity of the beads were restored (Figure 9).

The initial number of viable cells present in each bead was 1 × 10^6^. After 5 treatment cycles, cell viability in each bead was 5 × 10^5^. Cell leakage from the beads was measured spectrophotometrically at 620 nm. O.D. value for cell leakage was 0.1 for five cycles, but cell leakage increased to 0.5 from the 6th cycle onwards. Each treatment cycle was repeated by washing the spent medium and resuspending the beads in fresh buffer with freshly added 0.2% TCE. 

**Figure 10:**
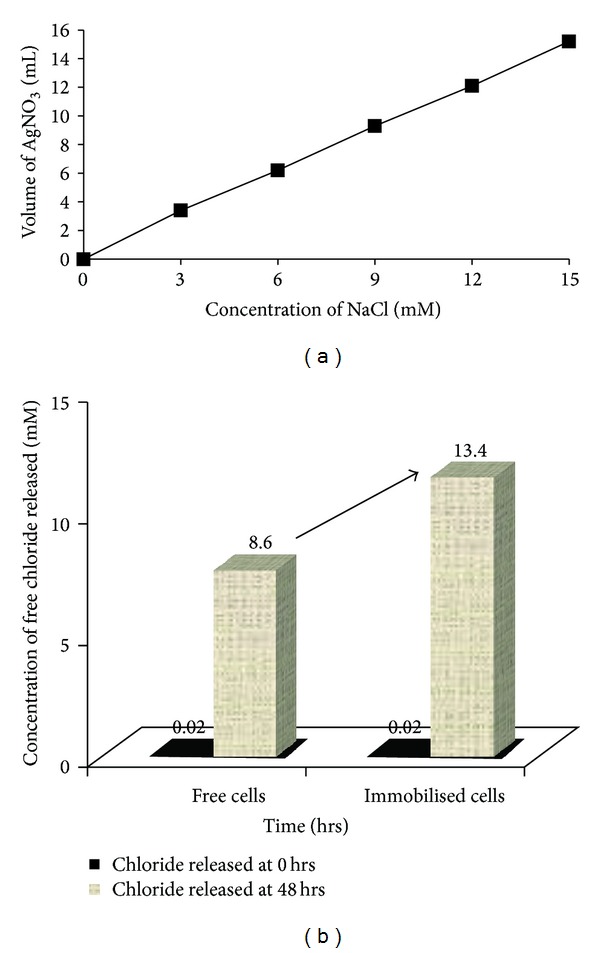
(a) Standard curve of chloride estimation by titration with 10 mM silver nitrate. (b) Concentration of free chloride released by mineralisation of TCE by the PM102 isolate before and after immobilisation, as plotted from the standard curve. Data shown for the first treatment cycle.

The beads could be reused 8 times. After cell immobilisation, significant increase in TCE degradation was obtained (Figure 10(b)). Chloride released at 0 hours, that is, just after cell inoculation, minimal chloride (0.02 mM), was detected in the solution of 0.05 mM Tris with 0.2% TCE. After 48 hours, chloride released by free cells was 8.6 mM while by immobilised cells was 13.4 mM, as calculated from the standard curve (Figure 10(a)). Reusability was measured in terms of TCE degradation capacity. The beads retained TCE degradation capacity for 8 treatment cycles but the free cells lost TCE degradation ability from the 2nd cycle onwards. Although some amount of cell leakage was found, persistence of viable cells in the beads was detected till the 8th treatment cycle. TCE is known to be degraded to TCE epoxide or chloral hydrates that are toxic for the cell. The calcium alginate layer protects the bacterial cells from the cytotoxic attack. This is a useful characteristic of the immobilised cell that can be exploited for bioremediation of TCE.

## 4. Conclusion

The *Stenotrophomonas maltophilia* PM102 isolate was found to utilise trichloroethylene as the sole carbon source. The bacterium does not have the capability to grow when toluene, benzene, and chloroform are present in the medium as the only carbon source, but 0.1% peptone is needed for growth. When phenol is present, even 0.1% peptone cannot help the bacterium to survive. The PM102 isolate could degrade TCE efficiently when it was present as the sole carbon source, but TCE degradation was enhanced in presence of benzene and chloroform. This indicates that some enzymes involved in the TCE degradation pathway may be common to the catabolic mechanism employed by this bacterium for the degradation of other chlorinated or aromatic contaminants. Further evidence confirming this hypothesis was obtained from the western blot where the antibody raised against TCE inducible proteins of PM102 strain also cross-reacted to toluene-, benzene- and chloroform, induced proteins obtained from the same strain. MALDI-TOF/MS identification of the common 35.14 kDa protein revealed that there is no prior record of this protein in the database. Further work involving purification and structural evaluation of this novel protein needs to be done. 

The theoretical amount of chloride released from 0.2% TCE is 15.22 mM, considering that all the TCE added remains in solution. In the titration experiment, chloride released by PM102 free cells was found to be 8.6 mM, after 48 hours, that increased considerably to 13.4 mM when PM102 cells were immobilised in calcium alginate. Thus, this bacterium could mineralise 0.2% TCE added initially to free chloride by 57% while after immobilisation TCE mineralisation by the same bacterium was enhanced to 88%. 

## Figures and Tables

**Table 1:** Detailed PMF data of the 35.14 kDa protein as matched with the hypothetical protein from *Bacteroides thetaiotaomicron* (UniProtKB/TrEMBL accession number: Q8A696).

Cal mass	Obs mass	±da	±ppm	Start seq.	End seq.	Sequence	Modification
1209.5933	1209.5203	−0.073	−60	305	314	DQIMAEYALR	
1547.7498	1547.6216	−0.1282	−83	136	146	ARYWYMIQCLK	Oxidation (M) [6]
1550.752	1550.745	−0.007	−5	64	76	LANDLKNDYPEMK	
1564.7286	1564.6475	−0.0811	−52	220	233	FLVEMGAGFAFMGR	Oxidation (M) [5, 12]
1855.8795	1855.8904	0.0109	6	1	15	MENQNHAFNYAYLLK	
2040.0193	2040.0731	0.0538	26	216	233	HIEKFLVEMGAGFAFMGR	
2134.9824	2134.9143	−0.0681	−32	28	45	AIYTANEEMLSMYWDIGK	
2150.9773	2150.9834	0.0061	3	28	45	AIYTANEEMLSMYWDIGK	Oxidation (M) [9]
2279.0723	2278.8794	−0.1929	−85	27	45	KAIYTANEEMLSMYWDIGK	Oxidation (M) [10]
3081.4648	3081.2236	−0.2412	−78	191	215	DPYIFDMLTFTEEYDERDIELGLIK	Oxidation (M) [7]

## References

[1] Van den Berg M, Birnbaum LS, Denison M (2006). The 2005 World Health Organization reevaluation of human and mammalian toxic equivalency factors for dioxins and dioxin-like compounds. *Toxicological Sciences*.

[2] Brüning T, Weirich G, Hornauer MA, Höfler H, Brauch H (1997). Renal cell carcinomas in trichloroethene (TRI) exposed persons are associated with somatic mutations in the von Hippel-Lindau (VHL) tumour suppressor gene. *Archives of Toxicology*.

[3] http://ntp.niehs.nih.gov/index.cfm?objectid=72016262-BDB7-CEBA-FA60E922B18C2540.

[4] Dual cure Low-solvent silicone pressure sensitive adhesives.

[5] Dees C, Askari M, Henley D (1996). Carcinogenic potential of benzene and toluene when evaluated using cyclin-dependent kinase activation and p53-DNA binding. *Environmental Health Perspectives*.

[6] Budavari S (1996). *The Merck Index: An Encyclopedia of Chemical, Drugs, and Biologicals*.

[7] International Agency for Research on Cancer (IARC) - Summaries & evaluations: chloroform. http://www.inchem.org/documents/iarc/vol73/73-05.html.

[8] Chang M-K, Voice TC, Criddle CS (1993). Kinetics of competitive inhibition and cometabolism in the biodegradation of benzene, toluene, and *p*-xylene by two *Pseudomonas* isolates. *Biotechnology and Bioengineering*.

[9] Fries MR, Forney LJ, Tiedje JM (1997). Phenol- and toluene-degrading microbial populations from an aquifer in which successful trichloroethene cometabolism occurred. *Applied and Environmental Microbiology*.

[10] Juteau P, Côté V, Duckett M-F (2005). *Cryptanaerobacter phenolicus* gen. nov., sp. nov., an anaerobe that transforms phenol into benzoate via 4-hydroxybenzoate. *IJSEM*.

[11] Rehfuss M, Urban J (2005). Rhodococcus phenolicus sp. nov., a novel bioprocessor isolated actinomycete with the ability to degrade chlorobenzene, dichlorobenzene and phenol as sole carbon sources. *Systematic and Applied Microbiology*.

[12] Hilge-Rotmann B, Rehm H-J (1990). Comparison of fermentation properties and specific enzyme activities of free and calcium-alginate-entrapped *Saccharomyces cerevisiae*. *Applied Microbiology and Biotechnology*.

[13] Kim MK, Singleton I, Yin C-R, Quan Z-X, Lee M, Lee S-T (2006). Influence of phenol on the biodegradation of pyridine by freely suspended and immobilized *Pseudomonas putida* MK1. *Letters in Applied Microbiology*.

[14] Chen X-A, Xu Z-N, Cen P-L, Wong WKR (2006). Enhanced plasmid stability and production of hEGF by immobilized recombinant *E. coli* JM101. *Biochemical Engineering Journal*.

[15] Mukherjee P, Roy P (2012). Identification and characterisation of a bacterial isolate capable of growth on trichloroethylene as the sole carbon source. *Advances in Microbiology*.

[16] Moss MS, Rylance HJ (1966). The Fujiwara reaction: some observations on the mechanism. *Nature*.

[17] Bradford MM (1976). A rapid and sensitive method for the quantitation of microgram quantities of protein utilizing the principle of protein dye binding. *Analytical Biochemistry*.

[18] Thiede B, Höhenwarter W, Krah A (2005). Peptide mass fingerprinting. *Methods*.

[19] Xu J, Bjursell MK, Himrod J (2003). A genomic view of the human-*Bacteroides thetaiotaomicron* symbiosis. *Science*.

[20] Crossman LC, Gould VC, Dow JM (2008). The complete genome, comparative and functional analysis of *Stenotrophomonas maltophilia* reveals an organism heavily shielded by drug resistance determinants. *Genome Biology*.

[21] United States National Institute for Occupational Safety and Health, Centres for Disease Control and Prevention Workplace safety and health topics. http://www.cdc.gov/niosh/topics/organsolv/.

